# Papillary muscle involvement in acute and chronic myocardial infarction: an MRI study using multi-contrast delayed enhancement pulse sequence

**DOI:** 10.1186/1532-429X-11-S1-O26

**Published:** 2009-01-28

**Authors:** Yuesong Yang, Kim Connelly, Jay Detsky, Gideon Paul, Graham A Wright, Alexander J Dick

**Affiliations:** Sunnybrook Health Sciences Centre, Toronto, ON Canada

**Keywords:** Mitral Regurgitation, Papillary Muscle, Blood Pool, Wall Motion Abnormality, Mitral Valve Regurgitation

## Introduction

The papillary muscle (PM) is an integral component of the mitral valve apparatus. Acute or chronic myocardial infarction (MI) with PM ischemia is a primary factor leading to the occurrence of mitral regurgitation, with associated substantial morbidity and mortality [1, 2]. PM-MI is also a source of ventricular arrhythmia in these patients [3]. Although DE-MRI using IR-FGRE can detect PM-MI, its accuracy is primarily limited by poor contrast between left ventricular (LV) blood pool and infarcted myocardium.

## Hypothesis

We hypothesize that multi-contrast delayed enhancement (MCDE) imaging will improve the identification of PM-MI in patients with acute and chronic MI, compared to conventional IR-FGRE imaging.

## Methods

Cardiac DE-MRI studies using both MCDE and IR-FGRE in patients with MI were reviewed. Twenty-three patients (21 males, 2 females, average age of 62 ± 10 years old; 5 acute MI within 7 days, 18 chronic MI > 4 weeks) met the diagnostic criteria of PM-MI, as outlined below. All studies were performed on a 1.5 T GE Signa HDx system (GE Healthcare, Milwaukee, WI), which included a short-axis oblique (SAO) and two or four chamber SSFP studies. Both IR-FGRE and MCDE covering the whole LV in SAO were performed 10–20 minutes after double-dose bolus injection of Gd-DTPA. For IR-FGRE, the TI varied from 200 to 300 ms, depending on the null point of healthy myocardium. For MCDE, a segmented SSFP readout is used following an inversion pulse, providing 20 cardiac-phase-resolved images at varying effective TIs [4]. The in-plane resolution was 1.5 × 1.5 mm for both techniques.

PM-MI was considered if the following criteria were satisfied in the IR-FGRE or MCDE images: (1) the increased signal intensity of PM was similar to that of adjacent hyper-enhanced infarct segments; (2) the hyper-enhanced PM region was limited to the PM area defined by pre-contrast SSFP. The contrast between blood pool and hyper-enhanced LV infarct was rated as excellent (3), good (2) or fair (1) based on their differentiation.

## Results

**Figure 1:**
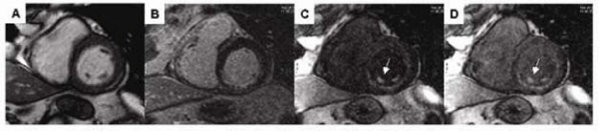
**PM involvement in a subject with RCA territorial infarction**. Pre-contrast SSFP image (A) depicted the morphology of PM. IR-FGRE image (B) did not show the PM-MI, while MCDE (C and D) clearly demonstrated the infarcted PM (arrows).

Based on the standard AHA 17-segment model [5], all patients with PM-MI demonstrated wall motion abnormalities and DHE involving multiple coronary artery territories. Of these 23 patients, 13 studies demonstrated primarily involvement of the territories of the RCA (8 patients) and/or LCX (5 patients) and 10 involved the territories of LAD with some LCX involvement. Although IR-FGRE and MCDE determined the presence and extent of LV MI equally well, better contrast scores were achieved in MCDE (2.9 ± 0.3) compared to IR-FGRE (1.6 ± 0.8, P < 0.001). MCDE clearly demonstrated PM-MI in all cases (100%, 23/23). However, only 39% (9/23) could be visualized in the corresponding IR-FGRE images (Figure 1), which displayed poor contrast between blood pool and infarcted myocardium. The multi-contrast capability of MCDE facilitates an improved differentiation between blood pool and infarct. This is especially important for the detection of PM-MI since a large of portion of PM extends within the LV blood pool and does not attach to the solid LV wall [6]. Moreover, the MCDE sequence provided cine images that also facilitated the simultaneous appreciation of wall motion abnormalities in the region of MI. Mitral valve regurgitation with mild or moderate degree was noted on four- and/or two-chamber SSFP scans in 52% (12/23) of patients.

## Conclusion

MCDE imaging provides better contrast between blood pool and infarcted myocardium, thus improving the determination of PM-MI that may help identify patients in whom the significant mitral regurgitation may affect morbidity and mortality.
